# Emergency open cholecystectomy is associated with markedly lower incidence of postoperative nausea and vomiting (PONV) than elective open cholecystectomy: a retrospective cohort study

**DOI:** 10.1186/1471-2482-10-6

**Published:** 2010-02-12

**Authors:** Jeffrey M East, Derek IG Mitchell

**Affiliations:** 1 Department of Surgery, Cornwall Regional Hospital, Montego Bay, Jamaica; 2 Department of Surgery, Radiology, Anesthesia and Intensive Care, University Hospital of the West Indies, Mona, Kingston 6, Jamaica

## Abstract

**Background:**

During a previous study to define and compare incidence risks of postoperative nausea and vomiting (PONV) for elective laparoscopic and open cholecystectomy at two hospitals in Jamaica, secondary analysis comparing PONV risk in elective open cholecystectomy to that after emergency open cholecystectomy suggested that it was markedly reduced in the latter group. The decision was made to collect data on an adequate sample of emergency open cholecystectomy cases and further explore this unexpected finding in a separate study.

**Methods:**

Data were collected for 91 emergency open cholecystomy cases identified at the two paricipating hospitals from May 2007 retrograde, as was done for the 175 elective open cholecystectomy cases (from the aforementioned study) with which the emergency cases were to be compared. Variables selected for extraction and statistical analysis included all those known, suspected and plausibly associated with the risk of PONV and with urgency of surgery.

**Results:**

Emergency open cholecystectomy was associated with a markedly reduced incidence risk of PONV compared to elective open cholecystectomy (6.6% versus 28.6%, P < 0.001). The suppressive effect of emergency increased after adjustment for confounders in a multivariable logistic regression model (odds ratio 0.103, P < 0.001). This finding also identifies, by extrapolation, an association between reduced risk of PONV and preoperative nausea and vomiting, which occurred in 80.2% of emergency cases in the 72 hour period preceding surgery.

**Conclusions:**

The incidence risk of postoperative nausea and vomiting is markedly decreased after emergency open cholecystectomy compared to elective open cholecystectomy. The study, by extrapolation, also identifies a paradoxical association between pre-operative nausea and vomiting, observed in 80.2% of emergency cases, and suppression of PONV. This association, if confirmed in prospective cohort studies, may have implications for PONV prophylaxis if it can be exploited at a sub-clinical level.

## Background

During a previous study to define and compare the risks of postoperative nausea and vomiting (PONV) for elective laparoscopic and open cholecystectomy in a Jamaican hospital population [1], data were collected on a number of emergency open cholecystectomy cases. Exploratory secondary analysis suggested that the risk of PONV after emergency open cholecystectomy was significantly less than after elective open cholecystectomy. The decision was made to further explore this unexpected finding in a separate study on an adequate sample of emergency open cholecystectomy cases.

A significantly decreased risk of PONV after emergency open cholecystectomy is likely to be related to some inherent property or specific effect of acute cholecystitis and biliary colic (the usual indications for emergency cholecystectomy), such as emetogenesis or acute inflammation and physiologic stress, or to preoperative treatment, since, on the face of it, there does not appear to be any known confounders (measured and unmeasured) which could explain this effect. It is unlikely that the mechanism of PONV suppression in this scenario involves receptor blockade, which is the predominant mechanism of action of most drugs currently used for PONV prophylaxis [2,3]. Prospective confirmation of PONV reduction in emergency cholecystectomy cases after appropriate multivariable risk adjustment would therefore seem to demand proposition of an alternate mechanism of PONV suppression with attendant implications for an unconventional pharmacologic approach to PONV prophylaxis.

Systematic review of the literature failed to reveal any prior studies comparing PONV risk after emergency versus elective surgery in general and after emergency versus elective cholecystectomy in particular, nor was any study on the possible effect of pre-operative nausea and vomiting on PONV risk encountered. Biedler et al [2], no doubt suspecting that pre-operative vomiting could be a confounder or effect modifier, specifically excluded such patients in their study of interventions to reduce PONV risk.

In this retrospective cohort study, the incidence risks of PONV after elective and emergency open cholecystectomy at two major hospitals in Jamaica are compared, by bivariate as well as multivariable logistic regression techniques. Plausible reasons for the observed difference in risk and implications for alternate pharmacological approaches to PONV prophylaxis are discussed.

## Methods

The study was approved by the separate Ethics Committees of the Western Regional Health Authority and the Faculty of Medical Sciences of the University of the West Indies in Jamaica.

Applying the risk of PONV for elective open cholecystectomy of 29% observed among 175 cases from the aforementioned study [1], the number of cases of emergency open cholecystectomy needed to achieve 80% power to detect a 10% risk as significant at the 5% level is 54 (Epi Info Version 3.3.2.). Cases were operations in which open cholecystectomy, elective or emergency, was the primary operation and in which the primary disease being treated was cholelithiasis. Emergency cases were defined as those who had surgery performed on or before the elective operating list following emergency admission to hospital for biliary colic or acute cholecystitis. Exclusions included operations in which cholecystectomy was incidental or secondary to another major procedure, such as common bile duct exploration or laparotomy for pancreatitis, cholecystectomy performed for disease other than cholelithiasis (eg, gallbladder cancer) and emergency cholecystectomy for acalculus cholecystitis. Cases of laparoscopic cholecystectomy were also excluded, whether successfully completed or converted to the open operation, because the laparoscopic approach was only rarely used to treat emergency cases at both hospitals and therefore including laparoscopic cholecystectomy cases in either comparison group could have introduced potentially significant selection bias.

Emergency cases were identified by perusing operations registers at the Cornwall Regional Hospital (CRH) in Montego Bay and University Hospital of the West Indies (UHWI) in Kingston from May 2007 retrograde, as was the method used to identify the previously collected 175 elective cases [1], until the requisite number was surpassed. Cases could only be classified and included (or excluded) after retrieval and perusal of the records as the operations register did not necessarily indicate urgency of surgery (most emergency cases were performed on the elective list following admission and would therefore not appear in the operations register as an emergency).

Variables selected for extraction included known, suspected and plausible risk factors for PONV. History of previous PONV or motion sickness, a known risk factor for PONV, was not selected because it was rarely recorded. Variables extracted from the records were hospital, age, sex, major systemic illness, estimated body mass index, current smoking status, use of pre-surgery or intraoperative nasogastric tube and when removed (recovery room or ward), pre-medication, PONV prophylaxis, induction with propofol, addition of succinylcholine as supplementary relaxant, volatile anesthetic agent, reversal of anesthesia, duration of anesthesia, peri-operative antibiotic, total narcotic analgesia from pre-medication to 24 hours after end of anesthesia, PONV on operating table (that is, after end of anesthesia but before and during transfer to recovery room) or recovery room, PONV on ward, time to first tolerated oral intake, vomiting or nausea in the 72 hour period preceding surgery and use of narcotic analgesia before surgery (excluding opioids used for pre-medication). A case of PONV was any patient manifesting retching or vomiting or reporting nausea within 24 hours of the end of general anesthesia, as defined by others [4]. The relevant information was identified by perusing the doctors' notes, anesthetic and recovery room charts and the nurses' notes. Data was extracted unto a pre-coded form and then entered into a STATA (Version 9) database for statistical analysis.

Summary statistics and analyses include frequency of PONV (with confidence intervals) in elective open cholecystectomy and emergency open cholecystectomy at either and both hospitals combined as well as chi-squared test of difference in proportions affected by PONV in each comparison group. Variables were then selected, as possible confounders, for stepwise inclusion in a multivariable logistic regression model of effect of urgency of surgery on PONV risk if they were individually associated with either PONV risk or urgency of surgery at a P-value of < 0.15 by t-test or chi-squared test as appropriate. Possible interaction between urgency of surgery and hospital in their effect on PONV risk was examined in a separate model for the purpose of determining the legitimacy of pooling the results from both hospitals for the statistical analysis. No interactions were examined in the final regression model.

## Results

Data were collected on 175 cases of elective open cholecystectomy, 150 from CRH (May, 2002 to May, 2007) and 25 from UHWI (March, 2001 to May, 2007), and 91 cases of emergency open cholecystectomy, 41 from CRH (January, 2002 to May, 2007) and 50 from UHWI (February, 2001 to May, 2007). At both institutions, a small number of case records identified from the operations registers either could not be located or were in circulation at the time of data retrieval and therefore could not be classified. The range of missing records that might possibly have satisfied inclusion criteria after classification is 0-4%(6/156) and 0 for elective and emergency open cholecystectomy respectively at CRH and 0-17%(5/30) and 0-6%(3/53) for elective and emergency open cholecystectomy respectively at UHWI, the upper limit of each range being the total number of records unavailable. Since this small number of potential case records appeared to be missing at random (either misplaced by filing clerks or sent off to clinics) and therefore unlikely to result in any systematic selection bias by their omission, no extraordinary attempt was made to locate them.

Table 1 illustrates the distribution of independent variables. Three continuous variables, namely age (coded as > or ≤ 50 years), duration of anesthesia (coded as > or ≤ 1.5 hours) and total opioid dosage (coded as > or ≤ 175 mg) were dichotomized to facilitate statistical analysis. The variable "PONV prophylaxis" represents patients given dimenhydrinate with each injection of narcotic. No patient received PONV prophylaxis in the conventional sense, by way of administration of an antiemetic independent of narcotic analgesia prior to initiation of anesthesia, and, in particular, none received a serotonin HT3 receptor antagonist for prophylaxis. None of the patients suffering pre-operative vomiting appeared to have received anti-emetic treatment in hospital.

**Table 1:** Distribution of independent variables by hospital and urgency of open cholecystectomy

	Hospital			
	**CRH**		**UHWI**	
	Emerg	Elect	Emerg	Elect
**Number of cases**	41	150	50	25
**Age**- Mean(Range)	49.1(8-81)	43.7(20-78)	42.7(20-82)	47(20-81)
**Sex**- No.(%) female	35(85.4%)	136(90.7%)	46(92%)	21(84%)
**Current non-smoker**- No.(%)	39(95.1%)	144(96%)	48(96%)	21(84%)
**Systemic illness- **No.(%)	24(58.5%)	65(43.3%)	20(40%)	12(48%)
**Estimated BMI- **No. obese(%)	20(48.8%)	70(46.7%)	28(56%)	14(56%)
**NG Tube**- No.(%)	36(87.8%)	113(75.3%)	40(80%)	5(20%)
**Premedication- **No.(%)	9(22%)	129(86%)	2(4%)	9(36%)
**PONV Proph**- No.(%)	7(17.1%)	23(15.3%)	48(96%)	24(96%)
**Induct. with Propofol-** No.(%)	10(24.4%)	11(7.3%)	26(52%)	18(72%)
**Relaxant (Sux)**- No.(%)	25(61%)	52(34.7%)	29(58%)	0(0%)
**Halothane**- No.(%)	38(92.7%)	148(98.7%)	46(92%)	21(84%)
**Reversal**- No.(%)	31(75.6%)	107(71.3%)	42(84%)	14(56%)
**Duration anesth**- Mean(range)	1.56(0.5-6.17)	1.47(0.75-3.58)	2.31(0.92-4.25)	1.81(0.92-2.92)
**Antibiotic**- No.(%)	40(97.6%)	135(90%)	49(98%)	17(68%)
**Total opioid- **Mean(range)	240(30-500)	182(50-400)	329(140-600)	358(175-610)
**Time to first oral intake**- Mean/(range)	36.54(10.5-240)	24.95(7-68)	37.53(6.75-96)	23.6(3.75-47)
**Vomit < 72 hours pre-surgery** - No.(%)	32(78.1%)	0(0%)	41(82%)	0(0%)
**Opioid < 72 hrs pre-surgery** (excludes pre-med)- No. (%)	11(26.8%)	0(0%)	12(24%)	0(0%)

Current non-smoker = never smoked or stopped more than 6 months earlier.

Obese = estimated BMI ≥ 30.

Ponv proph = PONV prophylaxis. No patient received PONV prophylaxis in the conventional way. The group reported here received dimenhydrinate 25-50 mg with each dose of opioid.

All patients received a non-depolarizing muscle relaxant but the group referred to here also received a dose of the depolarizing relaxant succinylcholine.

Most patients received the volatile inhalation agent Halothane, as shown here. The others received isoflurane or sevoflurane.

Reversal was with neostigmine and atropine.

**All **times are in hours.

Total opioid dosage from immediately preoperative to 24 hours postoperative is expressed in meperidine equivalency units in milligrams, where fentanyl 100 μgms = meperidine 100 mgs = morphine 10 mgs.

Table 2 illustrates the distribution of postoperative nausea and vomiting by urgency of surgery and hospital as well as the results of bivariate intra-hospital and combined comparison of the risk of PONV by urgency of surgery and the odds ratio for the effect of emergency on PONV risk from the crude bivariate logistic regression model. At both hospitals, separately and then after pooling, the risk of PONV after emergency open cholecystectomy is significantly less (at the 5% level) than the risk after elective open cholecystectomy. Pooling by urgency of surgery (emergency, elective) was justified on the basis that (a) there was no statistically significant difference between the age structure of cases within each category of operation by hospital (P = 0.07 and 0.26 by t-test for emergency and elective open cholecystectomy respectively) nor between the gender structure (P = 0.31 for both emergency and elective open cholecystectomy), (b) there was no reason to suspect that the indications for emergency open cholecystectomy (including failure of resolution of acute cholecystitis or biliary colic, severity of acute cholecystitis, concomitant diabetes mellitus or immunosuppression, stone wedged in Hartman's pouch on ultrasound examination, departmental policy for treatment of biliary colic or acute cholecystitis and availability of operating time) were differentially distributed between the hospitals and (c) most importantly, there was no interaction between hospital and urgency of surgery in their effect on PONV risk, neither after stratification by hospital (P = 0.69, test of homogeneity of odds ratios) nor after testing the interaction parameter in a crude logistic regression model including only these three parameters and the interaction term (P = 0.68, Wald test).

**Table 2:** Distribution of postoperative nausea and vomiting by hospital and urgency of open cholecystectomy.

Hospital	Acuity	No. Cases	PONV OR/RR No.(%)	PONV Ward No.(%)	PONV No.(%)(95% CI)
CRH	Emerg	41	0(0%)	3(7.3%)	3(7.3%)(1.5 - 19.9%)
	Elect	150	8(5.3%)	41(27.3%)	42(28%)(21 - 35.9%)
UHWI	Emerg	50	1(2%)	3(6%)	3(6%)(1.3 - 16.5%)
	Elect	25	0(0%)	8(32%)	8(32%)(14.9 - 53.5%)

TOTAL	Emerg	91			6(6.6%)(2.5 - 13.8%)
	Elect	175			50(28.6%)(22 - 35.9%)

OR/RR = Operating room/Recovery room

CI = Confidence interval

For risk of PONV after emergency versus elective open cholecystectomy at CRH, P = 0.006 (chi-squared test).

For risk of PONV after emergency versus elective open cholecystectomy at UHWI, P = 0.003 (chi-squared test).

For risk of PONV after emergency versus elective open cholecystectomy at both hospitals combined, P < 0.001 (chi-squared test).

Unadjusted odds ratio for effect of emergency on PONV risk = 0.176 (CI, 0.072 - 0.43, P < 0.001).

Variables not associated with PONV nor urgency of surgery (the main outcome and exposure variables) at P < 0.15 by bivariate analysis (chi-squared and t-test where appropriate) were excluded from the final logistic regression model, except for hospital which was kept despite absence of significant effect, as recommended for analysis of multicenter trials, to represent any effect from the different sample sizes at the two hospitals [5]. Variables thereby excluded from the regression model for lack of effect included estimated body mass index, systemic illness, age and current smoking status.

Urgency of operation (emergency) had a highly significant suppressive effect on PONV risk in the crude bivariate logistic regression model at the 5% level (odds ratio 0.176; CI, 0.072-0.43; P < 0.001) (Table 2.). Nasogastric tube, injection of dimenhydrinate with narcotic analgesia, induction with propofol, succinylcholine as supplementary relaxant, Halothane as volatile anesthetic agent, reversal with neostigmine (and atropine), duration of anesthesia, peri-operative antibiotic and total opioid dosage from premedication to 24 hours post-anesthesia were all eliminated from the logistic regression model after stepwise forward inclusion because of lack of effect at P < 0.15. It was not reasonable to test the effect of time to first meal since the mean of this variable in both groups was equal to or greater than the 24 hrs during which PONV was assessed, indicating that early feeding could not have predisposed to PONV in this study population.

Table 3 displays parameters for the final multivariable logistic regression model. The only variables which demonstrated an effect on PONV risk at the 5% level of significance were urgency of surgery (emergency), female gender and pre-medication. Interestingly, the suppressive effect of emergency increased (odds ratio 0.103) after adjustment for the other two variables.

**Table 3:** The final multivariable logistic regression model for the effect of emergency on PONV risk.

Variable	Odds ratio (OR)	P-Value	95% CI for OR
Urgency of surgery (emergency)	0.103	< 0.001	0.036 to 0.297
Hospital (UHWI)	0.804	0.638	0.324 to 1.994
Female gender	10.451	0.025	1.34 to 81.743
Premedication	0.413	0.042	0.177 to 0.968

The suppressive effect of "emergency" on PONV risk increased from odds ratio 0.176 in the crude bivariate, logistic regression analysis to 0.103 after adjustment for the variables shown.

The effect of preoperative nausea and vomiting and of preoperative opioid administration cannot be assessed independently of urgency of operation in this logistic regression model because both variables are perfectly correlated with emergency cholecystectomy (that is, only emergency cases suffered preoperative nausea and vomiting or were treated with opioids separate from premedication). However, preoperative opioid administration was not associated with PONV among patients having emergency cholecystectomy (P = 1, Fisher's exact test) and therefore cannot explain the effect of emergency. Performing a similar analysis for the effect of preoperative nausea and vomiting within the emergency group is probably not legitimate since we have no way of knowing whether the emetogenic effect of acute gallbladder disease might not be active at a sub-clinical level in those who did not vomit or report nausea in the 72 hours preceding surgery or whether vomiting which might have occurred prior to this arbitrarily selected 72 hour period might not have a similar negative association with PONV. Because of this multicollinearity, the high incidence of pre-operative vomiting within 72 hours of surgery in emergency cases (80.2%) translates into an association between preoperative nausea and vomiting and suppression of PONV.

## Discussion

The markedly decreased risk of PONV after emergency open cholecystectomy compared to the elective operation is an unexpected finding. The well known shortcomings of retrospective studies notwithstanding, the profundity of the suppressive effect of emergency after adjustment for plausible confounders, the fact that the magnitude of the effect is similar at both hospitals separately and the considerable increase in power achieved by including 91 cases (greater than 95% power) in the emergency group rather than the 54 cases required to achieve 80% power all provide strong support for its validity.

The retrospective design might have resulted in underestimation of the true, absolute risks of PONV. It is unlikely that many instances of vomiting were missed or unrecorded, since recovery room charts have a section specifically for recording occurrence of PONV and since ward nurses meticulously record episodes of vomiting and retching (both hospitals are training institutions for nurses) as well as patient complaints (expected to capture most clinically significant episodes of nausea), but some episodes of nausea might not have been reported to nurses, particularly if transient and mild. Any reporting bias, however, would be expected to affect both comparison groups equally at each hospital and should therefore not change the odds ratio for the effect of emergency at each hospital. No variable, measured or unmeasured, was identified which could plausibly explain any increased likelihood of underreporting of PONV in emergency cases, either by patients or nurses and anesthetists. If underreporting of PONV in emergency cases was a significant source of error, it would be extremely unlikely to manifest itself to almost the same degree at both hospitals. The remarkable similarity between the odds ratios for the effect of emergency at each hospital (0.2 at CRH and 0.14 at UHWI, P = 0.82) certainly favors the more plausible explanation that there is some variable common to emergency cases which explains the effect observed.

Selection bias also appears to be minimal, or at least unlikely to explain the profound effect observed. More than 90% of emergency cases over the data collection period were performed by the open method, even by surgeons who offer laparoscopic cholecystectomy as standard of care for elective cases. If there was any selection bias, it would therefore apply more to the small emergency laparoscopic group than to the emergency open group, hence the decision to exclude those emergency cases performed laparoscopically. Elective open cases were also not subject to significant selection, being performed predominantly by surgeons who do not offer the laparoscopic approach in their repertoire. Having decided that the emergency comparison group should only include open cases, the elective comparison group also had to exclude laparoscopic cases to ensure a fair comparison.

Any error resulting from unavailable records should not be significant. The percentage of unavailable records was low at both hospitals and a significant proportion of those missing would have been ineligible for inclusion after classification (see exclusionary criteria above). It is unlikely that records would have been missing for reasons related either to the outcome (PONV), this being a transient phenomenon never requiring follow up, or to the main explanatory variable (urgency of surgery), there being no statistical association with systemic comorbidity nor any evidence of greater complication rate for either category of operation, which would require more intensive clinic follow up.

The bulk of the data for all groups was collected over a common time period (May, 2002 to May, 2007) so there is little opportunity for error resulting from changes over time in either the disease or its treatment. Any changes in anesthetic technique would have been captured among the variables extracted and adjusted for in the analysis.

Is it possible that the effect of emergency is shrouding the effect of some known or unknown confounder? Of several variables which were positively associated with emergency on bivariate analysis by chi-squared test (nasogastric tube, P < 0.01; administration of dimenhydrinate with opioid, P < 0.01; induction with propofol, P < 0.01; duration of anesthesia, P < 0.01; prophylactic antibiotic, P < 0.01; supplemental relaxation with suxamethonium, P < 0.01; non-use of premedication, P < 0.01) only relaxation with suxamethonium was even marginally associated with PONV (P = 0.049) and therefore none can explain the profound effect of emergency. The effect of smoking was not important in this study because of the very low frequency among cases. History of PONV or motion sickness, another established risk factor for PONV [4], could not be measured in this retrospective study but cannot explain the effect of emergency as it is unlikely to be of sufficiently high frequency and unlikely to be differentially distributed between the two groups compared, there being no known or plausible association with acute cholecystitis or biliary colic.

The reasonable inference therefore, is that the effect of emergency is due to some property or variable inherent and common to emergency cases (predominantly acute cholecystitis and biliary colic). In this regard, the profound emetogenic effect of these diseases, for which emergency cholecystectomy is performed, does not escape attention. Preoperative nausea and vomiting within 72 hours of surgery afflicted 80.2% of emergency cases and it would not be unreasonable to assume that it would have occurred prior to 72 hours pre-surgery in some of the other emergency cases. Additionally, this variable may well be operable at a sub-clinical level in those who did not overtly manifest it.

The other property that could plausibly explain the suppressive effect of emergency on PONV risk would be the non-specific effects of acute inflammation. If this is true, there should be a decrease in PONV risk after operations for acute inflammatory surgical disease in general, compared to elective surgery. Unfortunately, no such comparisons have been encountered in the literature to either support or refute this possibility. It is at least theoretically plausible that acute phase reactants and cytokines could blockade vomiting receptors or, more likely, induce those elements of the cytochrome P450 enzyme system in the liver which are responsible for degradation of the emetogenic agents used during anesthesia [6,7]. It appears, however, that the net effect of these endogenous chemicals on the CYP-450 system is suppression rather than stimulation [7].

The association between preoperative nausea and vomiting and PONV suppression identified in this study is intuitively paradoxical. However, sustained preoperative stimulation of the central vomiting centers could plausibly render them unresponsive during and immediately following surgery. This adaptive response has not been confirmed for the vomiting centers because of difficulties in creating an experimental model for nausea and vomiting [8], but if the vomiting center did not respond in this way, it would be relatively unique among neurological systems, most of which respond to sustained stimulation by becoming refractory to further stimulation or by upward resetting of the threshold for evocation of a response [9].

The association between preoperative nausea and vomiting and decreased risk of PONV identified herein is simulated by evidence indicating that cigarette smoking and preoperative nicotine patches, which are potent emetogens [10], are also associated with PONV suppression [4,11]. Sweeney [12] has suggested that the suppressive effect of smoking on PONV risk could plausibly be explained by sustained preoperative stimulation of the vomiting centers resulting in adaptation, but subsequently rejected this postulate on the basis that the emetogenic effect of smoking disappears rapidly in persistent smokers. But this development of tolerance for nausea by cigarette smokers may well reflect adaptation or upward resetting of the threshold of the vomiting centers in response to sustained stimulation, and, by extrapolation, could explain the apparent refractoriness of the vomiting centers in smokers to the emetogenic chemicals used during anesthesia. Sweeney favored another plausible mechanism of action, that the stimulatory effect of components of cigarette smoke on the CYP-450 system leads to enhanced metabolic degradation of emetogenic drugs used during anesthesia [12].

This study therefore identifies a powerful negative association between preoperative nausea and vomiting and PONV risk similar to the association between cigarette smoking or administration of nicotine patches and PONV suppression. The questions are whether adaptation of the vomiting centers is a plausible mechanism of action of cigarette smoking and nicotine patches and whether similar adaptation occurs in response to other emetogenic stimuli, such as biliary colic and acute cholecystitis. The association identified herein should be confirmed in a prospective cohort study, which need not restrict eligible cases to those in which open surgery is performed, but must include patients in which there is sustained preoperative nausea and vomiting. If confirmed, the implication would be that agents mimicking the emetogenic effect of the acute conditions of the gallbladder treated in our emergency cases, administered preoperatively and acting at a sub-clinical level, may be effective in reducing the risk of PONV.

## Conclusions

The incidence of postoperative nausea and vomiting is markedly decreased after emergency open cholecystectomy compared to elective open cholecystectomy (6.6% versus 28.6%, P < 0.001). The study, by extrapolation, also identifies a paradoxical association between pre-operative nausea and vomiting, observed in 80.2% of emergency cases, and suppression of PONV. This association, if confirmed in prospective cohort studies, may have implications for PONV prophylaxis if it can be exploited at a sub-clinical level.

## Competing interests

The authors declare that they have no competing interests.

## Authors' contributions

Both authors participated in the acquisition of data and revision of the manuscript. JME conceived of the study, determined the design, performed the statistical analysis, interpreted the data and drafted the manuscript. Both authors read and gave final approval for the version submitted for publication.

## Pre-publication history

The pre-publication history for this paper can be accessed here:

http://www.biomedcentral.com/1471-2482/10/6/prepub
